# Recognizing Biological Motion and Emotions from Point-Light Displays in Autism Spectrum Disorders

**DOI:** 10.1371/journal.pone.0044473

**Published:** 2012-09-06

**Authors:** Evelien Nackaerts, Johan Wagemans, Werner Helsen, Stephan P. Swinnen, Nicole Wenderoth, Kaat Alaerts

**Affiliations:** 1 Department of Biomedical Kinesiology, Group Biomedical Sciences, Research Center for Movement Control and Neuroplasticity, KU Leuven, Heverlee, Belgium; 2 Laboratory of Experimental Psychology, KU Leuven, Leuven, Belgium; 3 Neural Control of Movement Lab, Department Health Sciences and Technology, ETH, Zurich, Switzerland; Centre Hospitalier Le Vinatier (Bât. 452), France

## Abstract

One of the main characteristics of Autism Spectrum Disorder (ASD) are problems with social interaction and communication. Here, we explored ASD-related alterations in ‘reading’ body language of other humans. Accuracy and reaction times were assessed from two observational tasks involving the recognition of ‘biological motion’ and ‘emotions’ from point-light displays (PLDs). Eye movements were recorded during the completion of the tests. Results indicated that typically developed-participants were more accurate than ASD-subjects in recognizing biological motion or emotions from PLDs. No accuracy differences were revealed on two control-tasks (involving the indication of color-changes in the moving point-lights). Group differences in reaction times existed on all tasks, but effect sizes were higher for the biological and emotion recognition tasks. Biological motion recognition abilities were related to a person’s ability to recognize emotions from PLDs. However, ASD-related atypicalities in emotion recognition could not entirely be attributed to more basic deficits in biological motion recognition, suggesting an additional ASD-specific deficit in recognizing the emotional dimension of the point light displays. Eye movements were assessed during the completion of tasks and results indicated that ASD-participants generally produced more saccades and shorter fixation-durations compared to the control-group. However, especially for emotion recognition, these altered eye movements were associated with reductions in task-performance.

## Introduction

Autism Spectrum Disorders (ASD) refers to a set of complex, polygenetic neurodevelopmental disorders which are characterized by social and communication deficits, in addition to repetitive behavior and restricted interests [1]. In the past, psychological research has increasingly focused on the identification of the social deficits associated with ASD. In this context, it has been shown repeatedly that individuals with ASD differ from typically developed (TD) individuals in their visual perception of facial expressions of emotions [2], reflective of a deficit in *facial* emotion recognition [3].

However, facial expressions are not the only source of input for conveying emotionally relevant information. In every-day situations, other sources - such as the communicator’s body language or “*bodily kinematics*” - are equally important, especially when facial expressions are inconsistent or unavailable to the observer. In vision research, point-light displays (PLDs), representing biological motion solely by a set of small lights or markers attached to the major joints of an actor’s body, provide a widely adopted paradigm to investigate bodily motion perception [4]. In TD-subjects, PLDs have been shown to provide sufficient information for recognizing the gender of an actor [5], the activity in which he/she is engaged [4] and, even, the emotional state of the actor [6]–[7]. Yet, to date, only a few research groups explored whether obsevers with an ASD are different from TD-observers in biological motion or *bodily* emotion perception from PLDs and results are not entirely consistent [8].

Using a similar set of PLD-stimuli, Moore and colleagues assessed the ability to recognize a person’s actions, subjective states, emotions, and objects conveyed by moving PLDs in groups of low-functioning ASD-children [9], high-functioning ASD-children [10] and high-functioning ASD-adults [11]. Compared to controls, ASD-subjects were shown to have a reduced ability in verbally reporting the subjective states and emotions from the displayed point light animations, but no differences were revealed in reporting actions or objects. In study by Atkinson (2009), the ability of ASD-individuals to recognize actions or emotions from PLDs was readdressed using forced-choice paradigms [12]. Overall, results were replicated by showing ASD-related impairments in emotion recognition. However, in contrast to the earlier studies, the ASD-group also revealed deficits in labeling the displayed actions from PLDs [12]. Consistent with this; two studies reported atypicalities in ASD-subjects on forced-choice tasks requiring the identification of ‘a person’ in a series of human PLDs of familiar activities (‘biological motion’) or phase-scrambled versions of the same PLD (‘non-biological motion’) [13]–[14]. Blake (2003) found that 8–10 year old children with ASD were specifically impaired in this task, while being similar in their performance on another visual discrimination task [13]. Moreover, this study also revealed a relationship between autism severity and the ability to recognize biological motion. In a replication of this task, Annaz (2010) adopted a developmental approach by testing children across the age range from 5 to 12 years. Although comparable performances were revealed at the earliest age tested, performance was shown to increase with age only in the TD-group not in the ASD-group [14]. Consistent with this are findings from a preferential looking paradigm in two-year old toddlers, indicating that only TD-children demonstrated a clear looking preference for ‘biological’ PLDs, whereas toddlers diagnosed with autism did not [15]. By contrast however, no accuracy differences were revealed for distinguishing biological from scrambled motion in adult subjects [18] (although reaction time differences were apparent). Also several studies with adult ASD-subjects failed to reveal any ASD-related impairments in either detection thresholds [16] or accuracy and reaction times [17] for identifying the movement direction of PLDs depicting a walking person.

As such, literature to date provides indications that (at least adult) individuals with ASD may be rather unaffected in perceptual processing of ‘human form’ (biological motion), but continue to exhibit specific impairments in higher-order judgments such as emotion processing from PLDs.

In the present study we adopt two recently developed PLD recognition tasks to address ‘biological motion’ and emotion recognition abilities in adults with an ASD. Similar to the tasks used by Freitag (2008) in adult subjects and by Blake (2003) and Annaz (2010) in children, the currently adopted *biological motion recognition task* involved the identification of ‘a person’ in a series of human PLDs (‘biological motion’) and scrambled versions of the PLD (‘non-biological motion’). Emotion recognition abilities were also tested using a forced-choice recognition paradigm (similar to the task adopted by Atkinson (2009)). Both tasks included reaction time measures in addition to accuracy assessments.

First, this design allowed us to re-explore whether adult ASD-subjects display specific problems with recognizing emotions from PLDs, or whether deficits are also evident on a more basic biological motion recognition task. Furthermore, since both the **emotion recognition** task and the basic **biological motion recognition** task were administered to the same group of ASD-subjects, the present study also allows a comparison of performance on the two tasks. Specifically, this new aspect allowed us to explore whether ASD-related alterations in emotion recognition potentially relate to or originate from more basic deficits such as biological motion perception impairments.

As a control, accuracy and reaction times were also assessed on two control reaction time tasks involving the identification of color changes in the moving point-light dots. These tasks were included to provide basic measurements of reaction times and to assess the ability of subjects to understand task instructions and attend to the presented stimuli. We additionally recorded **eye movements** during the completion of the different tests. Although exploratory, these data allowed us to test whether differences in eye movements are apparent and, more importantly, whether differential performance on the PLD recognition tasks potentially corresponds to differences in eye movements.

## Methods

### Ethics Statement

Written informed consent was obtained from all participants prior to the experiment, after explaining the study and its procedure. Consent forms and study design were approved by the local Ethics Committee for Biomedical Research at the KU Leuven in accordance to The Code of Ethics of the World Medical Association (Declaration of Helsinki). All potential participants who declined to participate or otherwise did not participate were not disadvantaged in any way by not participating in the study.

### Participants

Twelve adult ASD-subjects (7 males) and 12 TD-subjects (7 males) participated in the study (Table 1). The groups were group-matched for age, gender, VIQ, PIQ and FSIQ as summarized in Table 1. VIQ, PIQ and FIQ were calculated from the Ward 7-subtest short-form of the Wechsler Adult Intelligence Scale-III [19]–[21].

**Table 1:** Descriptive data.

	ASDMean (SD)	TDMean (SD)	Group comparison
N	12	12	
Gender (M:F)	7∶5	7∶5	
Age in years	34.9 (8.5)	31.5 (6.3)	t(22) = 1.11; p = .28
Verbal IQ	114.6 (16.6)	113.4 (14.8)	t(22) = .18; p = .86
Performance IQ	105.7 (20.1)	115.3 (15.8)	t(22) = −1.31; p = .21
FS IQ	111.5 (18.2)	115.5 (15.1)	t(22) = −.59; p = .56
SRS	99.4 (30.2)	37.2 (18.9)	t(22) = 6.05; p<.001

Inclusion criteria for the ASD group were: 1) a diagnosis of autistic disorder according to the DSM-IV (Diagnostic and Statistical Manual of Mental Disorders, fourth edition) criteria and 2) scores above 60 on the Social Responsiveness scale (SRS) [22]–[23].

The included ASD-participants had received a written diagnosis of Autistic disorder (n = 9) or Asperger’s syndrome (n = 3) from a multidisciplinary team of clinicians qualified according to the DSM-IV criteria [1]. The SRS was used to confirm or exclude the presence of substantial ASD symptoms in the patient and control groups respectively. Higher scores on the SRS indicate greater severity of social impairment. Control and ASD-participants scored significantly different on the SRS (Table 1) [t(22) = 6.05, p<.001]. Variations in age and FSIQ were not intrinsically correlated, or related to the SRS-scores [all, p>.1]. Due to technical problems, data on the *Emotion recognition test* were lost for one ASD and one control-participant.

### Stimuli

**Figure 1:**
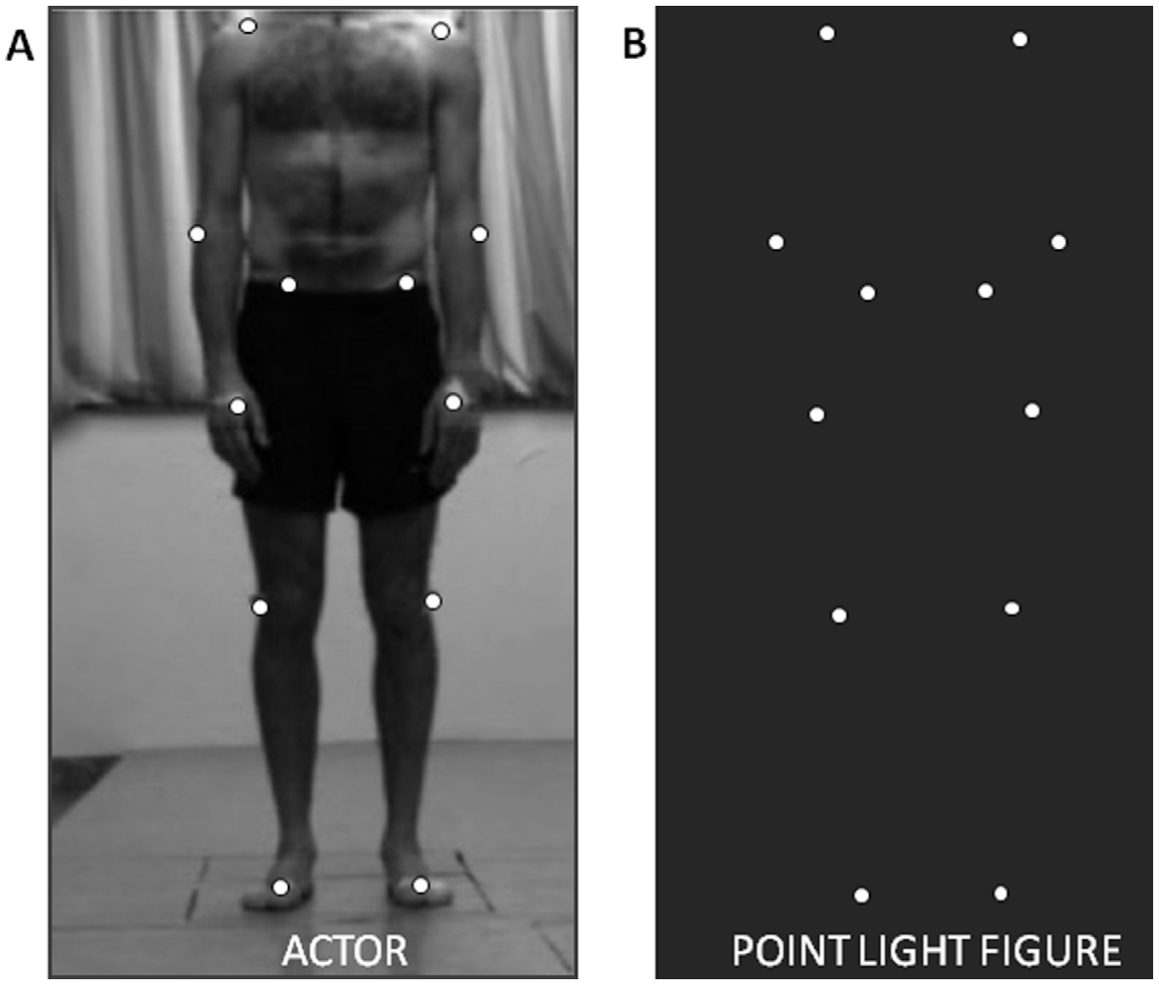
Exemplary point light display. (**A**) Photograph of the male actor with the 12 markers attached to the body and (**B**) the corresponding point light figure.

In all tests, stimuli consisted of moving point-light displays (PLDs) of a male and female actor. Stimuli were based on motion capture data as previously described [24]. In short, twelve reflective markers attached to the joints of the ankles, the knees, the hips, the wrists, the elbows, and the shoulders, were tracked using an eight-camera VICON system (capturing system measuring at 100 Hz, Oxford Metrics, Oxford, UK) (Figure 1A, 1B) (the actor in figure 1A has given written informed consent, as outlined in the PLoS consent form, to publication of his photograph). In the adopted movie files (duration 3 sec), marker positions were visible as twelve moving white spheres on a black background from three different viewpoints (front view (0°), side view (90°), intermediate view (45°)). The moving dots subtended 11×12 degrees visual angle at an approximate viewing distance of 50 cm. Each dot subtended 0.25 degrees. The presented PLDs portrayed persons performing one of three different actions: *walking*; *jumping* on the spot; or *kicking* a ball using the right leg. The emotional state of the actor in the PLDs could either be *neutral*; *happy*; *sad*, or *angry*.

For each of the adopted PLDs, a scrambled version was created which consisted of the same individual dots, undergoing the same local trajectories as in the intact PLDs, however with the initial starting position of the 12 individual dots randomly permutated to a different starting position. In the final PLDs, we chose not to include the marker on the head of the actors to avoid the observing subjects from relying predominantly on the position of this maker to judge the emotional state of the point light figure, or to decide whether the PLD presented a person or not.

For example movies, see Supporting Video S1, S2 and S3.

### Procedure

Participants completed two testing sessions; one consisting of the *Biological motion recognition test* and a *2-choice control-test*; the other consisting of the *Emotion recognition test* and a *4-choice control-test*. A brief description of the tests is provided below. A more detailed description and normative data on the adopted stimuli are provided elsewhere [24]. All stimuli (PLD-movies) were presented on a Tobii T120 Eye Tracker Screen which recorded eye movements at a constant rate of 120 Hz using five infrared lights integrated below the 17-inch screen (Tobii Technology AB, Danderyd, Sweden). Participants sat at a viewing distance of approximately 50 cm from the screen (note that subjects were free to make small trunk and head movements). Standardized instructions were provided verbally and on the monitor at the start of each test. In addition, a set of familiarization trials were presented and a simple 5-point eye-calibration was performed to verify point of gaze before each testing session.

A first testing session consisted of four alternating blocks of the *Biological motion recognition test* and the *2-choice control-test*. Each block consisted of 10 trials (PLD movies).

#### ‘Biological motion recognition test’

Subjects were presented with a series of PLDs that either depicted a person’s movements (‘biological motion’) or not depicted a person’s movements (‘scrambled’). Participants had to indicate as fast and accurate as possible whether the presented PLDs represented “a person” or “not a person”. The response options (person, no person) were indicated on the respective response buttons.

#### ‘2-choice color test’

In this test, subjects were presented with the same set of PLDs (‘biological motion’, ‘scrambled’) used in the *Biological motion recognition test* but one of the dots changed its color to either red or green at an unpredictable time point. Participants were instructed to indicate as fast and accurate as possible which color was shown by pressing different buttons on a keyboard (marked with red and green).

A second testing session consisted of three alternating blocks of the *Emotion recognition test* and the *4-choice control-test*. Each block consisted of 8 trials.

#### ‘Emotion recognition test’

In this test, each trial consisted of a ‘prime’-PLD, followed by a ‘target’-PLD. Participants were asked to indicate as fast and accurate as possible whether the presented point-light figure in the ‘target’-movie performed the displayed action in a different ‘emotional state’ compared to the point light figure in the ‘prime’-movie. The emotional state of the target could either be indicated as happier, sadder, angrier, or not different, from the prime. The four response options (happier, sadder, angrier, no difference) were indicated on the keyboard. Prime- and target-movies remained constant with respect to (i) the presented model (e.g. if the prime was male, also the target was male) and (ii) the type of action displayed (e.g., if the prime was a walking point-light figure, also the target was a walking point-light figure). On the other hand, the viewing perspective was always different between prime- and target-movies (e.g., if the prime was viewed from the front view, the target was presented either from the side view or the 45° view). The prime-movie always showed a point-light figure in the ‘neutral emotional state’, whereas the emotional state of the target point-light figure could either be happy, sad, angry or neutral.

#### ‘4-choice color Test’

This test was administered in two blocks. In the first block, stimuli were identical to the set of prime-target sequences used in the *Emotion recognition test*. In the second block, the scrambled versions of the prime-target sequences were presented. In the prime-movie, one of the moving white dots briefly (0.5 sec) changed color to either ‘red’ or ‘green’ at a random time point. In the target-movie, three of the moving dots changed color and participants had to indicate (as fast and accurate as possible) how many of the dots (0–1–2 or 3) changed to the same color as the dot in the prime-movie. The four response options (0–1–2 or 3) were indicated on the keyboard.

All participants completed the two test sessions in the same order, such that all subjects were comparably ‘naive’ on the nature of ‘intact’ versus ‘scrambled’ PLDs in the *Biological motion recognition test*.

### Data Analysis

#### Test performance

For each test, reaction times (RTs) and accuracy rates (% correct answers) were assessed using Tobii Studio 1.3.21 software (Tobii Technology AB, Danderyd, Sweden).

For the *Biological motion recognition test*, we also calculated the hit rate (responding “person” to a biological PLD), false alarm rate (responding “person” to a scrambled PLD) and the sensitivity index d’ (Table S1). RTs were considered as outliers and removed from the analysis when they exceeded Q3±1.5×(Q3–Q1) with Q1 and Q3 denoting the first and third quartile over the whole set of trials for each subject (Electronic Statistics Textbook, 2007, StatSoft, Inc. Tulsa). Following these criteria, only few trials were discarded from the RT-analyses [*Biological motion recognition test*: 3.12% of trials across all subjects] [*2-choice control-test*: 4.25%] [*Emotion recognition test*: 3.97%] [*4-choice control-test*: 5.52%]. The number of discarded trials was comparable for both groups (ASD and TD) on all tests.

#### Eye movements

To explore potential differences in eye movements between groups (ASD and TD), the number of saccades per second and the mean fixation duration were calculated over the whole set of trials of each test. Only the period lasting from the start of a trial (movie) until a response key was pressed was taken into account. A fixation was defined as the period of time when the eyes remained stationary within a fixation-radius of 58 pixels, or 1 degree of movement tolerance, for a period equal to, or greater than, 120 ms [25].

To ensure that both groups were engaged in the tasks and attended the presenting PLD stimuli, we calculated the percentage of time spent fixating on the displays (% On-scene Fixation Time) and the number of off-scene fixations (# Off-scene Fixations). As shown in Table S2, groups displayed similar amounts of on-scene fixating time as well as off-scene fixations. Only for the *2-choice control test*, the ASD-group fixated less on the PLD stimuli (reduced % On-scene Fixation Time, and higher number of Off-scene Fixations) (See Table S2).

#### Statistics

All statistics were calculated with Statistica 9.0 (StatSoft. Inc. Tulsa, USA).

Differences in performance (accuracy and RTs) and eye movements (saccades/second, fixation duration) were explored between groups (ASD and TD) and tests (experimental, control). For the RT data, repeated measures ANOVA analyses were conducted with group (ASD, TD) as the between-subjects variable and ‘test’ as the repeated-measures variable.

Group differences in accuracy, sensitivity index d’, saccades/second and fixation durations were tested using non-parametric Mann-Whitney U tests due to violations of the normality assumption (as indicated by Shapiro–Wilk tests). For the (non-normal) variables, we specifically explored whether group differences on the experimental tasks (*Biological motion recognition test, Emotion recognition test*) exist over and above group differences in the control tests (*2-choice control test, 4-choice control test*), by exploring group effects on the experimental/control ratio scores. Also Cohen’s d effect sizes were calculated to assess the size of group effects.

Multiple regression analyses were conducted to assess the relationship between performance on the *Emotion recognition test* and the *Biological motion recognition test* (corrected for performance on the control tests). We also explored the relationship between test performance (accuracy) and eye movements (saccades/second and fixation duration) (separately for each test) using non-parametric Spearman correlations (Table S3).

Additional multiple regression models were constructed to explore the extent to which the independent variables (i) SRS-scores, (ii) FSIQ, and (iii) age were related to test performance (dependent variable). Regression results of these models are summarized in Table S4.

## Results

### Group Differences in Accuracy

#### Biological motion recognition

**Figure 2:**
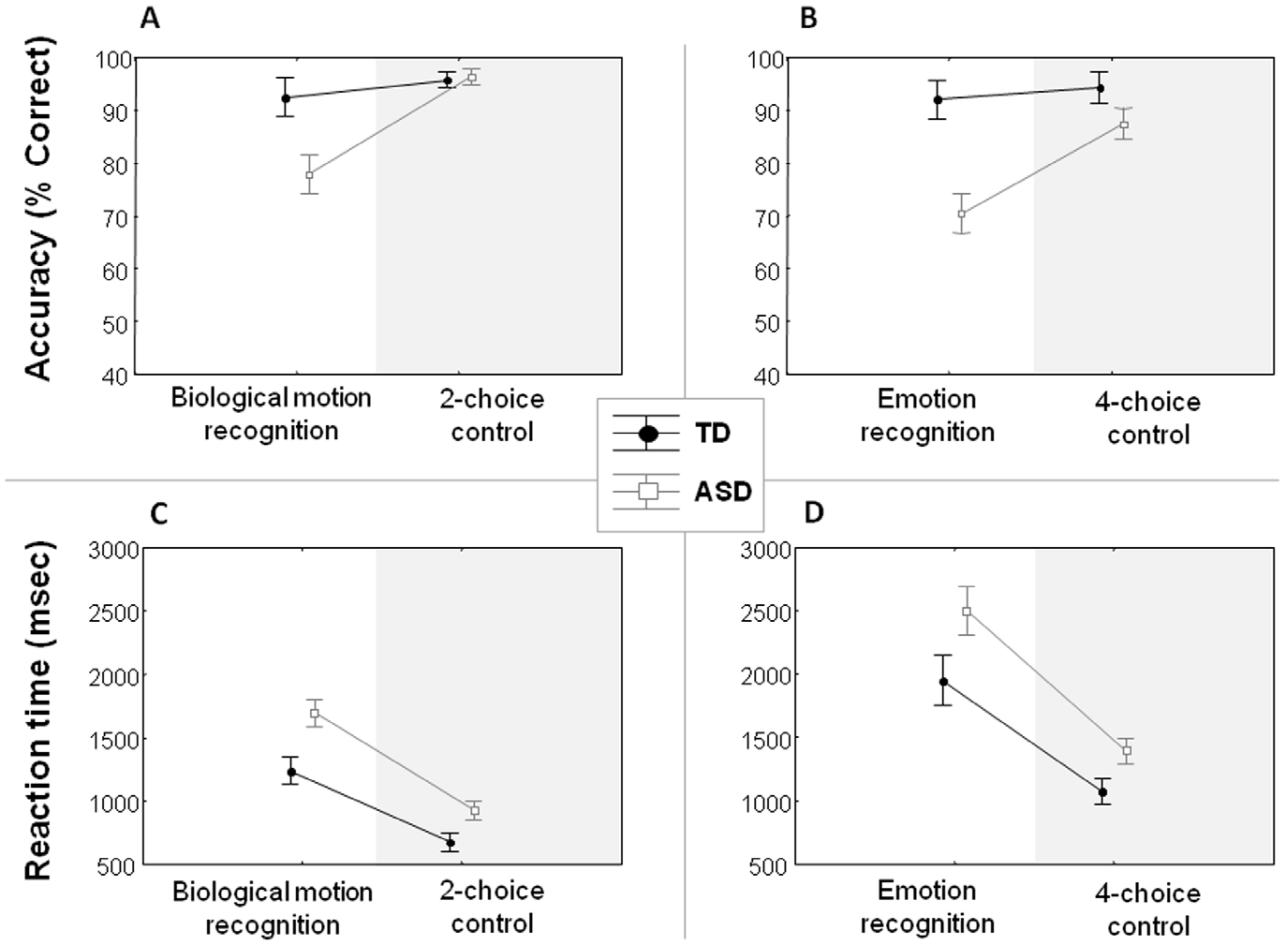
Test performance. Accuracy (% correct scores) (**A–B**), and reaction times (**C–D**) are displayed for all tests as a function of participant group (Autism spectrum disorders (**ASD**); Typically developed (**TD**)). Vertical lines denote ±standard error.

As a group, the TD-participants performed significantly more accurate compared to the ASD-group on the *Biological motion recognition test* as revealed by a significant group effect on the % correct answers [Z = 2.28, p = .022] [Cohen’s d = 1.16] (Figure 2A). No such difference in accuracy was revealed between groups on the *2-choice control-test* [Z = −1.01, p = .312] [Cohen’s d = −.11] (Figure 2A). Importantly, the accuracy group difference on the *Biological motion recognition test* persisted over and above group-related differences on the *2-choice control test*, as revealed by a significant group effect on the [experimental/control] ratio scores [Z = 2.68, p = .007] [mean (SE) ASD:.81 (.044)] [mean (SE) TD:.97 (.023)].

Additionally, for each participant, we calculated the hits (responding “person” to a biological PLD) and false alarms (responding “person” to a scrambled PLD) and used these measures to compute the d', an unbiased measure of sensitivity (Table S1). Similar to the accuracy results, the difference between groups in sensitivity index d' was statistically significant [Z = 2.19, p = .028].

#### Emotion recognition

TD-participants were shown to be significantly more accurate compared to the ASD-participants in recognizing the displayed emotions from the presented PLDs as revealed by an effect of group on the % correct answers [Z = 3.02, p = .002] [d = 1.77] (Figure 2B). Accuracy scores were comparable across groups on the *4-choice control-test* [Z = 1.54; p = .123] [d = .71] (Figure 2B). Also here, the accuracy group difference on the *Emotion recognition test* persisted over and above group-related differences on the *4-choice control test*, as revealed by a significant group effect on the [experimental/control] ratio scores [Z = 2.66, p = .008] [ASD:.81 (.044)] [TD:.98 (.038)].

### Group Differences in Reaction Times

#### Biological motion recognition

The ‘group × test’ ANOVA analysis on the reaction time data revealed a significant main effect of ‘group’ [F(1,22) = 10.29, p = .004]; indicating that reaction times were generally higher for the ASD-group compared to the TD-group (irrespective of the type of test). Also a main effect of ‘test’ was revealed, indicating higher reaction times on the *Biological motion recognition test*, compared to the *2-choice control test* [F(1,22) = 107.54, p<.001]. The ‘group x test’ interaction failed to reach significance [F(1,22) = 2.55, p = .124] (Figure 2C).

#### Emotion recognition

Reaction times were generally higher for the ASD-group compared to the TD-group (irrespective of the type of test) as revealed by a main effect of ‘group’ [F(1,20) = 4.55, p = .045]. Also a main effect of ‘test’ was revealed, indicating higher reaction times on the *Emotion recognition test*, compared to the *4-choice control test* [F(1,20) = 138.93, p<.001]. The ‘group × test’ interaction failed to reach significance [F(1,20) = 1.87, p = .187] (Figure 2D).

### Group Differences in Eye Movements

#### Biological motion recognition. Saccades/second

**Figure 3:**
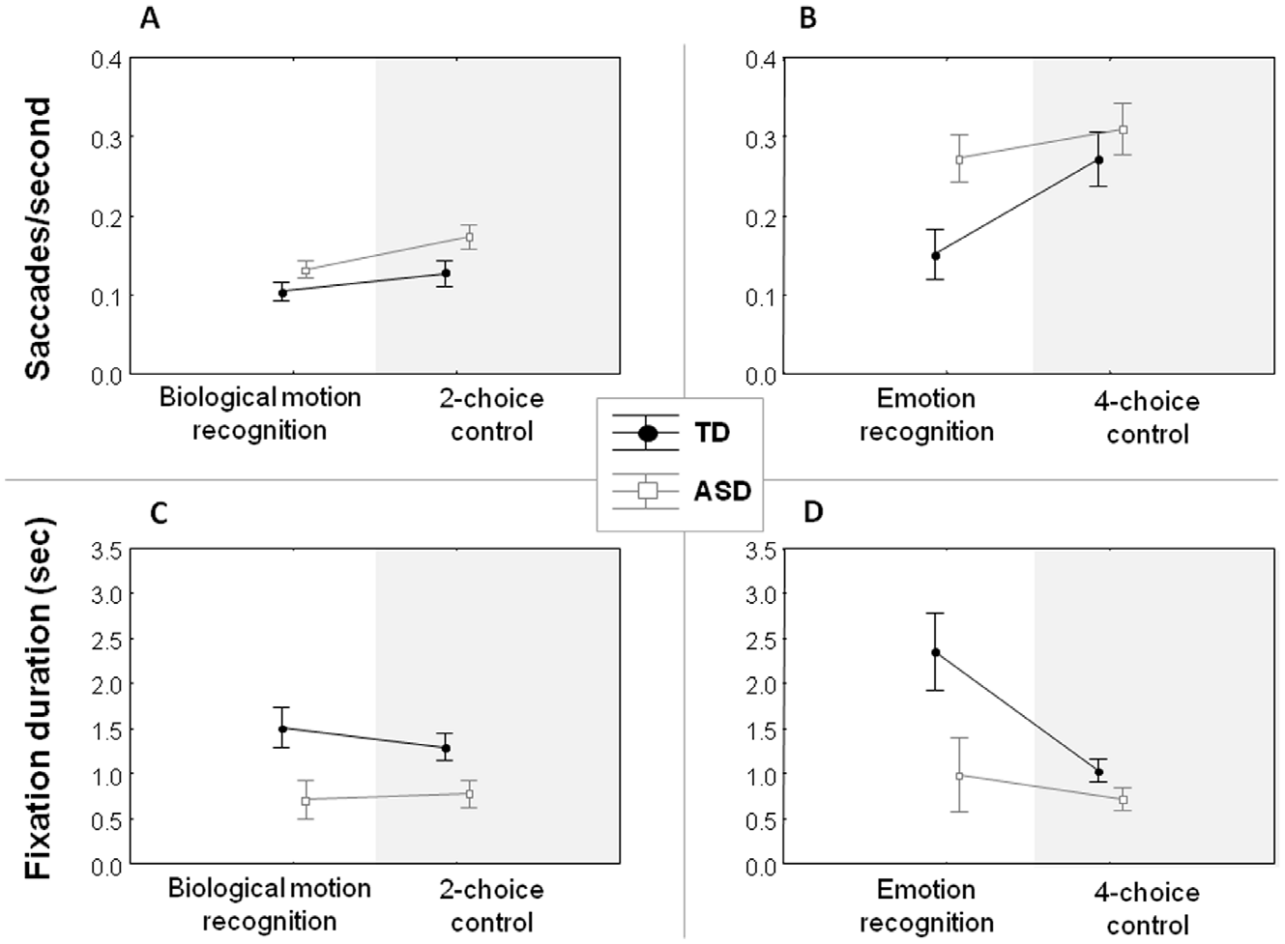
Eye movements. Saccades/second (**A–B**), and fixation durations (**C–D**) are displayed for all tests as a function of participant group (Autism spectrum disorders (**ASD**); Typically developed (**TD**)). Vertical lines denote ±standard error.

ASD-participants produced more saccades per second compared to the TD-group both on the *Biological motion recognition test* [Z = −1.94, p = .05] and the *2-choice control-test* [Z = −2.12, p = .034] (Figure 3A). Effect sizes even tended to be higher for the *2-choice control-test* [d = −3.01] compared to the *Biological motion recognition test* [d = −2.53]. No ‘group x test’ interaction effect was revealed from the [experimental/control] ratio scores [Z = .462, p = .644] [ASD:.81 (.092)] [TD:.85 (.077)].

#### Fixation duration

Also the duration of a fixation was shorter for the ASD-group compared to the TD-group both on the *Biological motion recognition test* [Z = 2.12, p = .033] and the *2-choice control-test* [Z = 2.31, p = .021] (Figure 3C). Effect sizes were comparable for both tests [d>3.4]. Also here, no ‘group x test’ interaction effect was revealed from the [experimental/control] ratio scores [Z = .585, p = .558] [ASD:.98 (.09)] [TD: 1.14 (.137)].

### Emotion Recognition

#### Saccades/second

Only on the Emotion recognition test, ASD-participants produced more saccades per second compared to the TD-group [Z = −2.5, p = .012] [d = −3.99]. No such group difference was revealed on the *4-choice control-test* [Z = −1.3, p = .19] [d = −1.12] (Figure 3B). Moreover, a significant ‘group x test’ interaction effect was revealed from the [experimental/control] ratio scores [Z = −2.29, p = .02] [ASD:.91 (.103)] [TD:.63 (.138)], indicating that the group difference on the *Emotion recognition test* persisted over and above group-related differences on the *4-choice control test*.

#### Fixation duration

The duration of a fixation tended to be shorter for the ASD-group compared to the TD-group both on the *Emotion recognition test* [Z = 1.8, p = .07] and the *4-choice control-test* [Z = 1.86, p = .08] (Figure 3D), although differences failed to reach significance. The size of the group effect tended to be larger however for the *Emotion recognition test* [d = 3.16] compared to the *4-choice control-test* [d = 2.44]. No ‘group x test’ interaction effect was revealed from the [experimental/control] ratio scores [Z = 1.302, p = .19] [ASD: 1.32 (.171)] [TD: 2.13 (.473)].

### Relationship between Biological Motion and Emotion Recognition

We conducted a multiple regression analysis to test the extent to which the independent variables (i) *4-choice control* accuracy and (ii) *Biological motion recognition accuracy* predicted accuracy on the *Emotion recognition test* (dependent variable).

Biological motion recognition accuracy was shown to significantly predict emotion recognition accuracy [ß = .43, t(19) = 2.10; p = .048] (over and above the variance explained by accuracy on the *4-choice control test* [ß = .11, t(19) = .51; p = .613]) [Whole model: R^2^ = .201, F(2,19) = 2.39, p = .118].

Next, a second regression model was run, using the same independent variables plus the factor ‘group’ to explore whether the inclusion of ‘group’ would significantly increase the proportion of variance explained (R^2^). Overall, this analysis explores whether emotion recognition performance would be solely predicted by the more basic ability to recognize human forms (i.e., if all variance in emotion recognition accuracy was explained by group-related differences in biological motion recognition, we wouldn’t expect the factor ‘group’ to explain any additional variance). Importantly, the inclusion of the factor ‘group’ still significantly improved the overall model (explaining an additional 27.9% of variance), indicating that there were group-specific differences in emotion recognition that were not accounted for by more basic biological motion recognition abilities [R^2^-change = .279, p = .006] [R^2^ = .480, F(3,18) = 5.54, p = .007].

However, no significant interaction was revealed between ‘group’ and ‘biological motion recognition abilities’ in predicting emotion recognition accuracy [ß = .25, t(17) = .151; p = .882] [R^2^ = .481, F(4,17) = 3.93, p = .019] indicating that the relationship was not significantly different for each group (in both groups, higher biological motion recognition accuracy relates to higher emotion recognition accuracy).

Furthermore, we conducted the ‘reverse’ multiple regression analysis testing the extent to which the independent variables (i) *2-choice control* accuracy and (ii) *Emotion recognition* accuracy predicted accuracy on the *Biological motion recognition test* (dependent variable). Similarly, emotion recognition accuracy significantly predicted biological motion recognition accuracy [ß = .45, t(19) = 2.09; p = .049] (over and above the variance explained by accuracy on the 2-choice control test [ß = .12, t(19) = .55; p = .586]) [Whole model: R^2^ = .188, F(2,19) = 2.19, p = .138]. However, inclusion of the factor ‘group’ as a third regression did not significantly improve the overall model’s R^2^ [R^2^-change = .065, p = .225] [R^2^ = .253, F(3,18) = 2.04, p = .144], indicating that individual differences in emotion recognition already explained most of the group-related variance in biological motion recognition.

No specific relationship existed between reaction times on the biological motion and emotion recognition test as substantially high correlations were revealed between reaction times scores from all four tests (experimental and control) [Pearson, all, r>.623, p<.002].

### Relationship between Eye Movements and Test Performance

A specific aim of this study was to explore potential relationships between eye movements (saccades/second, fixation duration) and performance (accuracy) on the four different tests. Table S3 reports the Spearman correlation coefficients for all relationships (across all participants, and within the ASD- and TD-group separately).

Overall, significant correlations were only revealed for the *Emotion recognition test*.

**Figure 4:**
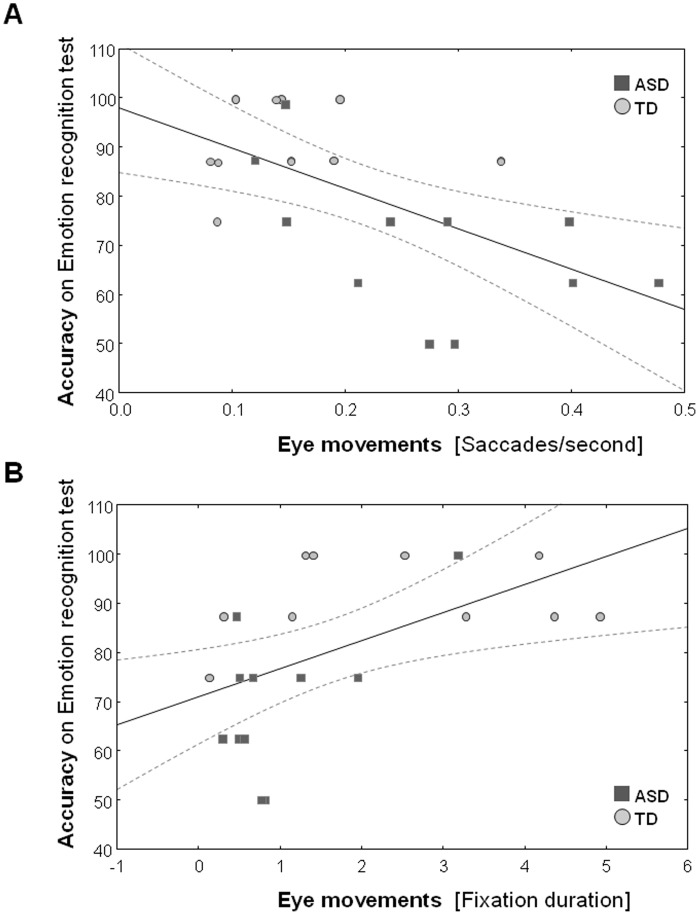
Across all participants, **figure 4** displays the linear fits for the relationship between accuracy on the *Emotion recognition test* (y-axis) and eye movements (x-axis) (saccades/second (A); fixation duration (B)). Dotted lines denote 0.95 confidence intervals.

Across all participants, higher accuracy scores on the *Emotion recognition test* were associated with making fewer saccades/second [r = −.60; t(20) = −2.56, p = .004] (Figure 4A) and longer fixation durations [r = .56; t(20) = 2.95, p = .008] (Figure 4B). For the *biological motion recognition test*, only the relationship between accuracy and saccades/second tended toward significance [r = −.38, t(21) = −1.901; p = .0711] (Table S3).

Within-group correlation analysis revealed that only within the ASD-group, the relationship between saccades/second and accuracy on the *Emotion recognition test* remained significant [r = .61; t(9) = −2.21 p = .05]

### Relationship between SRS-scores and Test Performance

We conducted an additional multiple regression analysis to explore the extent to which the independent variables (i) SRS-scores, (ii) FSIQ, and (iii) age would predict performance accuracy (dependent variable) on each of the four tests. Regression results are summarized in Table S4.

**Figure 5:**
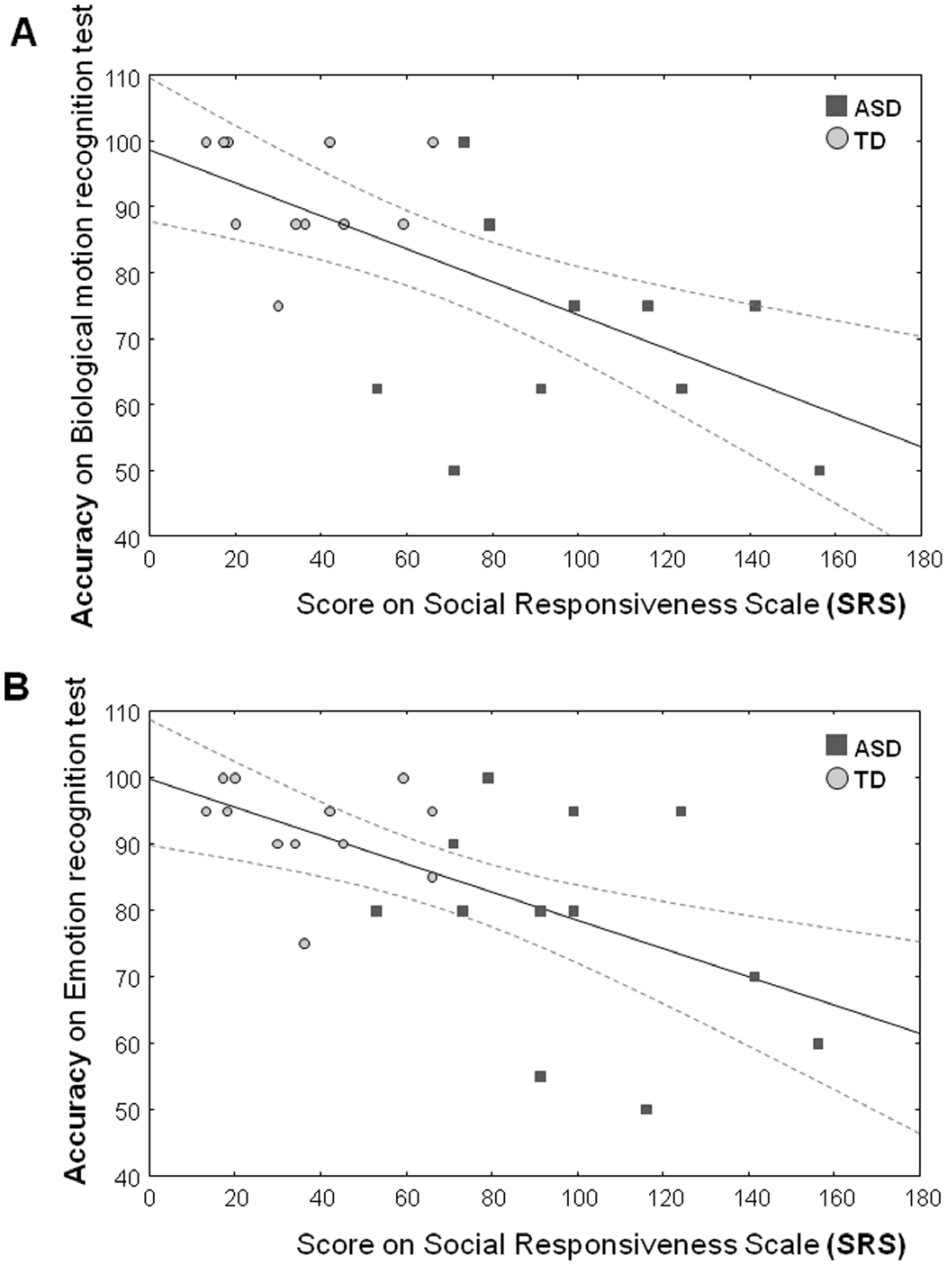
Across all participants, **figure 5** displays the linear fits for the relationship between scores on the social responsiveness scale (SRS) (x-axis) and accuracy (y-axis) on the *Biological motion recognition test* (A) and the *Emotion recognition test* (B). Dotted lines denote 0.95 confidence intervals.

Across all participants, only the **SRS-score** (not FSIQ or age) was shown to be a significant predictor for accuracy on the *Biological motion recognition test* [ß = −.53, t(20) = −2.85, p = .009] (Figure 5A); and accuracy on the *Emotion recognition test* [ß = −.47, t(18) = −2.89, p = .009] (Figure 5B) (indicating reduced performance with increasing SRS-score). No such relationship existed between SRS-scores and performance on the *2-* or *4-choice control-test* (Table S4).

Since group effects were shown to exist for the SRS-scores and accuracy scores of the *Biological motion* and *Emotion recognition test*, an additional correlation analysis was performed within the ASD-group alone. However for both tests, this analysis failed to reveal a significant correlation [Biological motion: Spearman Rank r = −.33, p>.1] [Emotion: r = −.10, p = .7].

## Discussion

The present study explored whether subjects with an autism spectrum disorder (ASD) showed specific performance deficits on tasks involving the recognition of ‘biological motion’ or ‘emotions’ from point-light displays (PLDs).

Results showed that typically developed (TD) participants were more accurate than ASD-subjects in recognizing biological motion or emotions from bodily kinematics depicted by the PLDs. Similar performance accuracies were revealed on two control-tasks involving the indication of color changes in the moving point-lights. Group differences in reaction times existed on all tasks, but effect sizes were higher for the biological and emotion recognition tasks compared to the control-tasks.

Biological motion recognition abilities were related to a person’s ability to recognize emotions from PLDs. However, ASD-related atypicalities in emotion recognition were not entirely accounted for by variations in basic human form recognition, providing indications for an ASD- specific deficit in attributing bodily emotions.

Also eye movements were assessed and results indicated that ASD-participants generally produced more saccades and shorter fixation durations compared to the TD-group. However, specifically for the emotion recognition test (not for the control-tests) this altered pattern of eye movements was related to test performance.

### Biological Motion and Emotion Recognition in ASD: Are Both Impaired?

Results from our ***Biological motion recognition*** *test* showed that adult ASD-subjects are less accurate than controls in identifying ‘a person’ in a series of intact PLDs or scrambled versions of the same PLDs. These findings are consistent with previous studies reporting ASD-related deficits on biological motion recognition tasks in ASD-children [13] and ASD-adolescents [26]. Moreover it extends previous studies revealing inconsistent results when testing adult ASD-subjects. Specifically, previous work failed to reveal any ASD-related impairments in either detection thresholds [16] or accuracy and reaction times [17] for identifying the movement direction of PLDs depicting a walking person. Also in a study by Freitag (2008), no accuracy differences were revealed for distinguishing biological from scrambled motion in adult subjects [18]. However, considering that substantially different tasks were adopted in our study and those by Saygin (2010) and Murphy (2009), it seems difficult to directly compare the reported results (the former studies used tasks that involved the identification of movement direction from PLDs, whereas in the present study, only basic recognition of biological motion was assessed). Also when relating our findings to those reported by Freitag (2008) on a similar ‘biological motion recognition task’, one can note apparent methodological differences in the nature of the adopted ‘scrambled PLD’. Specifically, in the present study, the intact versus scrambled PLDs only differed in the presence or absence of the global structure of a human body but not in the velocity profile of the individual moving points. In the scrambled PLDs used in the Freitag study, on the other hand, also the velocity profiles of the moving dots were replaced with a constant velocity, possibly lowering discrimination efforts between ‘intact’ and ‘scrambled’ motion and inducing ceiling performance (hence the lack of accuracy differences in this design) [18].

To date, a more consistent pattern of results emerged for emotion recognition. Findings from our ***Emotion recognition*** *test* showed ASD-related reductions in performance accuracy. These results largely replicate findings from previous studies also reporting aberrant emotion recognition in children [9]–[10] and adults [11]–[12]. Despite several methodological differences, strikingly similar patterns of ASD-related aberrations in emotion recognition are revealed from the forced-choice emotion recognition paradigms adopted by Atkinson (2009) [12]. In this former study, adult ASD-subjects were reliably less accurate in classifying bodily expressions of anger, happiness and disgust and marginally less accurate in classifying expressions of fear and sadness. Similarly, in the present study, also ‘happiness’, ‘anger’ and ‘sadness’ were adopted as main emotional states and also our study revealed very robust ASD-related impairments in emotion recognition.

In addition to accuracy differences, ASD-participants also showed increased reaction times to recognize biological motion or emotions from PLDs. However, general decrements in motor reaction times may have accounted – at least partly - for this effect, since group differences were also evident within two control reaction time tasks albeit less pronounced in terms of effect size. Differences in reaction times have been reported before on a related biological motion recognition task [18]. In terms of emotion recognition, the present study is the first to assess and report ASD-related differences in reaction times.

Across participants, performance on the *Biological motion* and *Emotion recognition test* was related to scores on the social responsiveness scale (SRS) [22], indicating reduced performance in subjects with more severe social impairments. However, when testing this relationship within the ASD-group alone, the correlation failed to reach significance. In this respect, results failed to extend previous findings from a study by Blake et al. (2003) with autistic children showing a significant relationship between severity of autistic symptoms and performance on a related biological motion recognition test [13].

To sum up, results from our study provide a unified picture of (adult) ASD-related deficits not only in emotion recognition abilities but also on more basic biological motion recognition tasks. These findings may contradict previous accounts which suggested that adult ASD-related difficulties in biological motion processing are restricted to the emotional domain.

### Relationship between Biological Motion and Emotion Recognition

For the first time, the adopted design allowed a direct comparison between biological motion recognition abilities and emotion recognition abilities in ASD-subjects. This comparison may not be trivial as previous studies from our lab and other labs already provided indications of a relationship between emotion and biological motion recognition abilities [24]–[27]. Data from the present study confirm these findings by showing that, across groups, a relationship existed between a subject’s ability to discern subtle emotional cues from PLDs and a subject’s ability to discriminate ‘intact biological motion’ from ‘scrambled motion’. When speculating on the direction of this relationship, it seems most intuitive to assume that an inability to recognize ‘low-level’ biological motion may preclude a ‘high-level’ attribution of emotional states to the perceived biological motion. Evidence exists however for a more parallel interpretation of the relationship, involving a substantial degree of cross-talk between these two processes. For instance, previous studies have shown that one’s ability to detect biological motion can be modulated by the emotional context of the stimulus, such that lower detection thresholds are observed for ‘angry’ biological motion compared to ‘happy’ biological motion [27]–[28]. Neri et al (2006) even demonstrated that the understanding of a social interaction between two agents embedded in point light display can enhance one’s ability to discriminate a human agent from the displays [29]. As such, also within the present study, the possibility cannot be ruled out that the ability to recognize emotional states in point light displays potentially contributed to one’s ability to decide on the biological nature of the presented point light stimuli.

An additional aim of this study was to address the extent by which variance in biological motion recognition abilities can predict the variance in emotion recognition, or whether group-identity (ASD or not) also contributes. Here, multiple regression analyses clearly indicated that although group-related difference in biological motion recognition are apparent and predictive of emotion recognition, they do not entirely account for all the group-related variance in emotion recognition. When considering the opposite relationship however, it appeared that group-related differences in emotion recognition were sufficient to explain most of the group-related variance in biological motion recognition. As such, although ASD-related deficits are apparent both in biological motion and emotion recognition, these data provide indications that ASD-specific deficits in high-level emotion recognition may exist over and above low-level deficits in biological motion recognition.

### Altered Eye Movements in ASD

On all tests, ASD-subjects tended to make more saccades and shorter fixation durations compared to control-subjects. However, only for PLD recognition tests (not for the control tests) these altered eye movements were related to test performance (significantly in terms of emotion recognition; tentatively in terms of biological motion recognition). To date, the majority of studies exploring eye gaze behaviour in autism have focused on ‘preferential looking tasks’ (for review see 30) which demonstrate that the attention of ASD-individuals is often abnormally distributed across social and nonsocial scenes, or social and nonsocial elements within a scene (e.g., reduced preference for the eyes and increased preference for the mouth when looking at facial expressions [31]–[33]). In the present study, increased saccade frequencies and shorter fixation durations in the ASD-group were revealed and these findings are in line with results from some previous studies assessing similar eye movement variables. In an early study by Kemner (1998) saccadic eye movements were evaluated in autistic children using an oddball task that consisted of frequent, rare, and novel pictures [34]. Autistic children were shown to make generally more saccades than normal children, especially for the frequently occurring stimuli. Also, unlike the normal group, their saccadic frequency did not depend on stimulus type [34]. Similar findings were revealed from a study by Keehn *et al*. (2009) [35] in which ASD-subjects displayed significantly shorter fixation durations during the completion of an Embedded Figures Test (EFT), i.e., a test in which subjects are instructed to identify the presence of simple ‘local’ figures embedded in a complex ‘global’ figure [35]. In general, individuals with ASD are known to excel on this task as a consequence of a ‘local’ (versus global) processing bias. This is a well-described feature within the Weak Central Coherence Theory of Autism (for review see [36]–[37]) positing that ASD-subjects focus more on local parts, whereas TD-individuals form a global percept before attending to the local elements of the global whole. As suggested before by Keehn *et al.* (2009), it can be hypothesize that the altered eye movements of the ASD-group (found rather robustly on all the adopted tests) are indicative of a general ‘local processing’ bias in ASD. Within this framework, it can be assumed that - irrespective of whether the task instruction relates to the recognition of features from PLDs, or to the detection of color changes in the moving dots - ASD-subjects may be attending more vigorously to the local trajectories of single point-light dots, instead of integrating the multiple dots to form a global gestalt. Especially for biological motion and emotion recognition, this altered ‘local’ processing strategy may have affected efficient task performance. In this respect, our eye gaze data may provide indications that the reported deficits in emotion and biological motion perception are - at least partly - related to a more general ‘global form from-motion’ deficit. In relation to the findings from our control tests however, it should be noted that the proposed ‘local processing bias’ may not be fully explanatory. Indeed, a firm interpretation of this account would not only predict deteriorated performance on the biological motion and emotion test, but would equally predict performance on the control tasks to be elevated in the ASD-group since these tasks require the *local* identification of color changes in one of the moving dots. This prediction was however not supported by our data (on both control tests, accuracies were comparable across groups and reaction times were higher in the ASD-group). Also the finding that group-related differences in emotion recognition exist over and above low-level differences in biological motion recognition may provide indications that - at least for emotion recognition - deficits cannot be entirely attributed to a ‘general local processing bias’, but additionally relate to an inability to decide on the social/emotional dimension of the point light stimuli. In either case, future research seems warranted to explore whether the altered gaze patterns are a general phenomenon or whether they are specific to particular (moving) stimuli or task-requirements. Indeed, although eye gaze alterations were found rather robustly in this and other studies [34]–[35], [38], also a number of studies exist [39], [40] that failed to reveal overall differences in the number of saccades or duration of fixations.

Finally, the possibility cannot be ruled out that other, more basic abnormalities in saccade generation may also contribute to altered eye gaze patterns in ASD. Several studies explored more elementary oculomotor abnormalities and although results are mixed, the majority of these studies indicated that basic features of saccadic eye movements (e.g., saccade accuracy, peak velocity, saccade suppression) may be abnormal in autism (e.g.,[41]–[42]) (for reviews see [43]–[44]).

### Brain Areas Underlying Biological Motion Perception

In relation to the PLD stimuli used in the present study, a number of studies consistently reported the brain’s action perception network (mirror neuron system (MNS)) and the superior temporal sulcus (STS) region to be involved in biological motion perception from PLD [45]–[48]. For example, Saygin (2004) reported that the frontal MNS becomes increasingly activated during PLD-perception [45]. Also recent data from a lesion study showed that the ability to recognize PLD-biological motion directly relies upon and requires neuronal resources that are part of the MNS [49]. Currently, there is a growing interest to explore the potential link between the MNS (and STS-region) in relation to the social cognition problems associated with ASD [50]–[51]. However, to date, only one study directly reported reduced activations in STS and the parietal MNS during the PLD perception in ASD [18].

To summarize, ASD-subjects showed reduced performances for recognizing biological motion or emotions from PLDs. Although performance on both tasks was related, reduced abilities to recognize emotional states in ASD were not entirely attributed to more basic deficits in human form recognition, suggesting an additional higher-order deficit in attributing bodily emotions. Altered eye movement behaviour in the ASD-group may be related to the ASD-related difficulties in emotion recognition from PLDs.

A limitation to the present study is the number of subjects included and the tested age range (only adult subjects were tested). Future studies should be conducted to generalize the reported findings to larger subject samples and broader age ranges (also including children and adolescents).

## Supporting Information

Table S1 **Table S1 displays the hit rate, false alarm rate and sensitivity index d’ separately for each group (ASD and TD).**
(DOCX)

Table S2 **For each group (ASD and TD), Table S2 summarizes the time spent fixating on the point light displays (% On-scene Fixation Time) and the mean number of off-scene fixations (# Off-scene Fixations).** Mean (SE) scores are displayed separately for each test (biological motion recognition, emotion recognition, 2-choice control and 4-choice control test).
(DOCX)

Table S3 **Table S3 summarizes the Spearman correlation coefficients testing the relationships between test performance (accuracy) and eye movements (saccades/second and fixation duration).** Correlation coefficients are reported separately for each test (Biological motion recognition, emotion recognition, 2-choice control and 4-choice control test).
(DOCX)

Table S4 **Table S2 summarizes the regression models testing the relationship between test performance (accuracy) (dependent variable) and the independent variables (i) SRS-scores (social responsiveness scale), (ii) FSIQ (full-scale IQ), and (iii) age.** Regression results are reported separately for each test (biological motion recognition, emotion recognition, 2-choice control and 4-choice control test).
(DOCX)

Video S1 **Exemplary movie of a point light display consisting of 12 moving white dots against a black background.** The point light display shows the female actor walking on the spot (neutral emotional state, side view).
(WMV)

Video S2 **Scrambled version of the point light display showed in Video S1.** It consists of the same individual dots, undergoing the same local trajectories as in the normal point light display, however with the position permutated between the 12 individual trajectories.
(WMV)

Video S3 **Exemplary movie of a ‘sad’ point light figure (female actor walking on the spot, front view).**
(WMV)
