# Supplementary material for: Recognizing Biological Motion and Emotions from Point-Light Displays in Autism Spectrum Disorders
Source: PLoS One. 2012 Sep 6;7(9):e44473. doi: 10.1371/journal.pone.0044473 (PMC3435310; doi:10.1371/journal.pone.0044473)
Supplement: Table S1 — displays the hit rate, false alarm rate and sensitivity index d’ separately for each group (ASD and TD). (DOCX) [file pone.0044473.s001.docx]

**Supplementary Table S1**

Table S1 displays the hit rate, false alarm rate and sensitivity index d' separately for each group (ASD and TD).

|  |  | | **ASD**  Mean (SE) | | **TD**  Mean (SE) |  |
| --- | --- | --- | --- | --- | --- | --- |
|  | Sensitivity d’ | | 1.83 (.280) | | 2.74 (.014) |  |
|  | Hit rate (responding “person” to a biological PLD) | | 78.3 (.073) | | 94.6 (.004) |  |
|  | False alarm rate (responding “person” to a scrambled PLD) | | 24.2 (.046) | | 15.4 (.038) |  |
|  |  |  | |  | | |
