# Supplementary material for: Recognizing Biological Motion and Emotions from Point-Light Displays in Autism Spectrum Disorders
Source: PLoS One. 2012 Sep 6;7(9):e44473. doi: 10.1371/journal.pone.0044473 (PMC3435310; doi:10.1371/journal.pone.0044473)
Supplement: Table S2 — For each group (ASD and TD), Table S2 summarizes the time spent fixating on the point light displays (% On-scene Fixation Time) and the mean number of off-scene fixations (# Off-scene Fixations). Mean (SE) scores are displayed separately for each test (biological motion recognition, emotion recognition, 2-choice control and 4-choice control test). (DOCX) [file pone.0044473.s002.docx]

**Supplementary Table S2**

For each group (ASD and TD), Table S2 summarizes the time spent fixating on the point light displays (% On-scene Fixation Time) and the mean number of off-scene fixations ( # Off-scene Fixations). Mean (SE) scores are displayed separately for each test (biological motion recognition, emotion recognition, 2-choice control and 4-choice control test).

|  | % On-scene Fixation Time | | | | | | |  |  | | # Off-scene Fixations | | | | | |  | |  |
| --- | --- | --- | --- | --- | --- | --- | --- | --- | --- | --- | --- | --- | --- | --- | --- | --- | --- | --- | --- |
|  | **ASD** | | **TD** | | | Group difference  (Mann-Whitney U) | | |  | **ASD** | | **TD** | | Group difference  (Mann-Whitney U) | | | |  |  |
| Biological motion recognition |  | 98.93 (0.59) | |  | 99.91 (0.06) | | Z= 1.33 | p= .18 |  | | 2.92 (1.29) | | 1.79 (0.69) | | Z= -1.10 | p= .27 | | | |
| Emotion recognition |  | 99.15 (0.30) | |  | 99.56 (0.21) | | Z= 0.81 | p=.41 |  | | 2.09 (0.72) | | 1.86 (0.53) | | Z= -0.58 | p= .56 | | | |
| 2-choice control |  | 98.72 (0.49) | |  | 99.93 (0.06) | | Z= 3.09* | p= .002* |  | | 3.75 (1.07) | | 2.04 (0.64) | | Z= -3.26* | p= .001* | | | |
| 4-choice control |  | 98.66 (0.62) | |  | 99.42 (0.29) | | Z= 0.31 | p= .75 |  | | 2.36 (0.89) | | 2.14 (0.58) | | Z= -0.46 | p= .64 | | | |
