# Supplementary material for: Recognizing Biological Motion and Emotions from Point-Light Displays in Autism Spectrum Disorders
Source: PLoS One. 2012 Sep 6;7(9):e44473. doi: 10.1371/journal.pone.0044473 (PMC3435310; doi:10.1371/journal.pone.0044473)
Supplement: Table S3 — summarizes the Spearman correlation coefficients testing the relationships between test performance (accuracy) and eye movements (saccades/second and fixation duration). Correlation coefficients are reported separately for each test (Biological motion recognition, emotion recognition, 2-choice control and 4-choice control test). (DOCX) [file pone.0044473.s003.docx]

**Supplementary Table S3**

Table S3 summarizes the Spearman correlation coefficients testing the relationships between test performance (accuracy) and eye movements (saccades/second and fixation duration). Correlation coefficients are reported separately for each test (Biological motion recognition, emotion recognition, 2-choice control and 4-choice control test).

|  |  | EYE MOVEMENTS | | | | | | | |
| --- | --- | --- | --- | --- | --- | --- | --- | --- | --- |
|  |  | **All** | |  | **ASD** | |  | **TD** | |
|  |  | Sac/sec | Fixation |  | Sac/sec | Fixation |  | Sac/sec | Fixation |
| ACCURACY | Biological motion recognition | -0.38 | -0.10 |  | -0.21 | -0.55 |  | -0.06 | -0.32 |
|  | Emotion recognition | -0.60* | 0.56* |  | -0.59* | 0.20 |  | 0.22 | 0.24 |
|  | 2-choice control | 0.17 | -0.40 |  | -0.06 | -0.48 |  | 0.23 | -0.09 |
|  | 4-choice control | -0.44 | 0.11 |  | -0.39 | 0.40 |  | -0.40 | -0.47 |
|  |  |  |  |  |  |  |  |  |  |
