# Supplementary material for: Recognizing Biological Motion and Emotions from Point-Light Displays in Autism Spectrum Disorders
Source: PLoS One. 2012 Sep 6;7(9):e44473. doi: 10.1371/journal.pone.0044473 (PMC3435310; doi:10.1371/journal.pone.0044473)
Supplement: Table S4 — Table S2 summarizes the regression models testing the relationship between test performance (accuracy) (dependent variable) and the independent variables (i) SRS-scores (social responsiveness scale), (ii) FSIQ (full-scale IQ), and (iii) age. Regression results are reported separately for each test (biological motion recognition, emotion recognition, 2-choice control and 4-choice control test). (DOCX) [file pone.0044473.s004.docx]

**Supplementary Table S4**

Table S2 summarizes the regression models testing the relationship between test performance (accuracy) (dependent variable) and the independent variables (i) SRS-scores (social responsiveness scale), (ii) FSIQ (full-scale IQ), and (iii) age. Regression results are reported separately for each test (biological motion recognition, emotion recognition, 2-choice control and 4-choice control test).

|  | Dependent variable | Independent variables | | | | |  |
| --- | --- | --- | --- | --- | --- | --- | --- |
|  |  | SRS |  | FSIQ |  | age | Whole model |
| ACCURACY | Biological motion recognition | ß=-.53, t(20)=-2.85  p=.009* |  | ß=.07, t(20)=.37  p=.717 |  | ß=-.20, t(20)=-1.15  p=.263 | R²=.40, F(3.20)=4.46  p=.015* |
|  | Emotion recognition | ß=-.47, t(18)=-2.89  p=.009* |  | ß=.30, t(18)=1.84  p=.082 |  | ß=-.30, t(18)=-1.95  p=.067 | R²=.59, F(3.18)=8.76  p=.001* |
|  | 2-choice control | ß=-.09, t(20)=-.399  p=.694 |  | ß=.12, t(20)=.56  p=.578 |  | ß=.39, t(20)=1.85  p=.078 | R²=.15, F(3.20)=1.22  p=.327 |
|  | 4-choice control | ß=-.19, t(18)=-.75  p=.462 |  | ß=.08, t(18)=.33  p=.742 |  | ß=.02, t(18)=.078  p=.939 | R²=.05, F(3.18)=.325  p=.807 |
|  |  |  |  |  |  |  |  |
